# Effect of management system and season on semen freezability in Jakhrana bucks

**DOI:** 10.14202/vetworld.2015.199-202

**Published:** 2016-02-20

**Authors:** Narendra Kumar, B. Rai, Showkat A. Bhat, S. D. Kharche, Chetna Gangwar, S. K. Jindal, Subhash Chandra

**Affiliations:** 1 Department of Livestock Production and Management, ICAR - National Dairy Research Institute, Karnal - 132 001, Haryana, India; 2 Department of Physiology Reproduction and Shelter Management, ICAR- Central Institute for Research on Goats, Mathura - 281 001, Uttar Pradesh, India

**Keywords:** freezability, Jakhrana, management system, season

## Abstract

**Aim:**

The objective of the study was to determine the effect of the management system (intensive and semi-intensive) and season (autumn and winter) on semen freezability in Jakhrana bucks.

**Materials and Methods:**

A total of 24 Jakhrana bucks of same body weight and age (BW=30 kg, age=1 year) were randomly allotted into two groups, *viz*., Group I (intensive system, 12 bucks) and Group II (semi-intensive system, 12 bucks). These two groups were statistically tested for their homogeneity with respect to age and BW. Semen was collected twice weekly using an artificial vagina during two seasons: autumn (September-November) and winter (December-February). A total of 240 semen samples (120 from each group and season) were evaluated for post-thaw motility (PTM), viability, abnormality, functional membrane integrity (hypo-osmotic swelling [HOS]) response and acrosomal integrity.

**Results:**

The mean values of PTM and acrosomal integrity of spermatozoa were significantly (p<0.01) higher in Group II as compared to Group I. The mean values of viability and abnormality were also differed significant (p<0.05) between groups. However, the mean values of HOS response were found non-significant (p>0.05) between groups. The season showed a significant effect on all parameters except viability and HOS response. The PTM and acrosomal integrity of spermatozoa were significantly (p<0.01) higher in winter as compared to autumn season. Abnormality of spermatozoa was significantly (p<0.05) lower in winter season.

**Conclusions:**

This study indicates that both management system and season influence semen freezability. The semen collected from bucks reared under the semi-intensive system and winter season showed better semen freezability characteristics.

## Introduction

The main constraint in any livestock production including goat is the scarcity of good breeding stock, as they are important to propagate the particular species. To improve the productive potential of goat the incorporation of superior germplasm into progeny is essential which is obviously possible with the use of outstanding sires.

Artificial insemination (AI) has made possible the effective use of best breeding males, thus greatly improving the genetic potential of breeding herds [1]. The ability to consistently select buck with high semen freezability is essential to obtain viable pregnancies following AI. Therefore, AI with frozen semen could be successfully used for preservation, *ex situ* conservation and propagation of goat germplasm [2]. The evaluation of sperm motility, viability, membrane and acrosomal integrity are essential criteria in the evaluation of the quality of a semen sample [3]. Moreover, semen quality and quantity is influenced by seasonal changes [4]. The influence of season has been reported on the fertilizing ability of spermatozoa after cryopreservation [5] which is an important limiting factor for the large scale application of AI with frozen semen.

Although information is available on the semen characteristics of several goat breeds but no information is available on management system and season on semen freezability in Jakhrana bucks. Moreover, the population of this breed is small which is localized only in few villages of Alwar district of Rajasthan. Considering that it is a good indigenous dairy breed, there is a need for its conservation. Thus, the objective of this study was to evaluate the effect of the management system and season on semen freezability in Jakhrana bucks. Such information will further useful for conducting successful AI in goat.

## Materials and Methods

### Ethical approval

The approval for the present study was obtained from the Institutional Animal Ethics Committee of Central Institute for Research on Goats.

### Location of study

The experiment was carried out in the Male Reproduction Lab of Central Institute for Research on Goats (CIRG), Makhdoom that is located at 27° N latitude, and 78° N E longitude on 176 m above the sea level.

### Experimental animals

A total of 24 Jakhrana bucks of same body weight and age (BW=30±2 kg, age=12±0.5 months) were randomly allotted into two groups *viz*. Group I (intensive system, 12 bucks) and Group II (semi-intensive system, 12 bucks). These bucks were already maintained into two different systems since weaning. These two groups were statistically tested for their homogeneity with respect to age and BW. All the animals were well grown and free from external parasites. The bucks under Group I were maintained under stall feeding conditions in the shed itself, and they were offered 500 g/head/day pelleted concentration mixture (crude protein [CP]: 18-19%; total digestible nutrients [TDN]: 65-70%) and 700 g/head/day green fodder comprising Lobia, Bajra and Jowar, besides available dry fodder *ad-libitum*. The bucks under Group II were grazed for 4-6 h daily on the available grazing area in the institute farm and supplemented with pelleted concentrate (CP: 18-19%; TDN: 65-70%) mixture at 400 g/head/day.

### Semen collection and evaluation

The bucks were trained to ejaculate in the artificial vagina (AV) before 15 days start of the experiment. A collection of semen was done with AV as per standard procedure followed at CIRG Makhdoom, during two seasons: Autumn (September to November) and winter (December to February). Immediately after collection the semen samples were placed in a water bath (37°C) and the initial evaluation was carried out within 10 min. The neat semen samples having mass motility of +3.0 and above, volume of 0.3 ml and above per ejaculate and free of coagulation after dilution were used for freezing. A total of 240 ejaculates (12 bucks×10 replicates from each group and season) were used for freezing and thawing. A dilution was done in tris buffer with 10% egg yolk (tris - 0.29 M, citrate - 0.1 M and fructose - 0.11 M, streptomycin – 100 mg and penicillin 1 lakh IU/100 ml, pH 6.8) and 6% glycerol to make final dilution rate of 1:10. An equilibration period of 4-h was allowed at 5°C in a cold handling cabinet. Then, the diluted samples were filled into 0.25 ml French medium straws and sealed with polyvinyl alcohol powder. The straws were then racked and exposed to liquid nitrogen (LN_2_) vapor by placing those 2 cm above the LN_2_ in a thermo box for 10 min before being plunged into LN_2_. Thawing of the straws was carried out individually at 40°C for 10 s in water bath for post-thaw sperm evaluation. After 4 h of equilibration, the straw was cut and a drop of semen (10 μl) of diluted semen was placed on warm (37°C) clean grease free slide and covered with cover slip and post-thaw sperm motility was examined under high power objective (×40) of phase contrast microscope (×400, magnification). Viability and abnormality were estimated by differential staining technique using Eosin-Nigrosin stain [6].The hypo-osmotic swelling test (HOST) was carried out as described by Gangwar *et al*. [7]. Acrosomal integrity of spermatozoa was assessed by Giemsa stain as described by Watson [8].

### Statistical analysis

The least square technique [9] was applied to investigate the fixed effects of management system (two classes; Intensive and semi-intensive) and season (two classes; autumn and winter) and their interaction on freezability parameters of semen. Differences among means were tested according to Duncan's multiple range test as modified by Kramer [10].

## Results and Discussion

The aim of the study was to reveal the effect of management system and season on semen freezability in Jakhrana bucks. The means along with their standard deviations and coefficient of variation of different semen freezability parameters used for analysis are presented in Table-1. The least square means of semen freezability parameters of both Groups (I and II) are given in Table-2. The mean post-thaw motility (PTM) and acrosomal integrity was significantly (p<0.01) higher in Group II. The result of PTM of both groups was almost similar to the findings of Tharasanit and Techakumphu [11] in Black Bengal goat but the PTM of semen in Group II bucks was lower than those reported in semi-intensively managed Jamunpari bucks [12]. Although it was higher in Group I than reported by Daskin *et al*. [13] in intensively managed Angora bucks. However, higher mean value of PTM than present finding was reported by Thakur *et al*. [14] in Chegu bucks maintained under semi-intensive system. The HOST response did not differ significantly between groups. The percent HOS responsive spermatozoa in Group I was lower (46.45±0.73) than reported by Priyadharsini *et al*. [15] who found 60.56±1.31 percent HOS responsive spermatozoa in Jakhrana bucks. However, the acrosomal integrity in the present study was higher than reported by Priyadharsini *et al*. [15]. The significance of an acrosomal cap is known to be a prerequisite for successful fertilization. Earlier researchers suggested that cryopreservation induces a reduction in acrosome integrity of frozen semen samples of goats [16]. Acrosome membrane intact sperm ranged between 40% and 70% in Canary [17] and Florida bucks [18]. It is presumed that loss of the acrosomal cap during freezing and thawing of buck sperm is similar to that demonstrated in bull sperm [19]. Tharasanit and Techakumphu [11] studied post-thaw semen of three Black Bengal bucks maintained under the intensive system, reported 83.7% intact acrosome which was much higher than present findings. However, the result of our study coincided with report of Al-Badry [20]. The mean value of percent viability and abnormalities of spermatozoa was significantly (p<0.05) higher in Group II and Group I, respectively. Cryopreservation adversely affects the cryosurvival of spermatozoa and under the best experimental conditions about 50% of motile sperm can survive the freeze-thaw process [21]. The present result of viability and abnormalities in both groups were lower than the finding of Ahmad *et al*. [22]. The mean abnormalities in semen of Group I bucks were almost similar to the finding of Apu *et al*. [23] in Black Bengal buck raised under intensive system, however, the percent viability of spermatozoa coincided with a report of Al-Badry [20].

**Table-1:** Descriptive statistics of different semen freezability parameters in Jakhrana bucks.

Parameters (%)	Mean	SD	Minimum	Maximum	CV (%)
PTM	41.47	7.72	25	55	18.19
Viability	49.27	7.25	32	64	14.65
Abnormality	12.84	3.55	4	24	27.43
HOS response	46.87	7.92	30	64	16.94
Acrosomal integrity	66.98	7.85	50	89	11.06

SD=Standard deviation, CV=Coefficient of variation, HOS=Hypo-osmotic swelling, PTM=Post-thaw motility

**Table-2:** Least square means±SE for effect of management system and season on different freezability parameters in Jakhrana bucks.

Parameters (%)	Number of sample	Management system		Season	
	
		Group I (intensive system)	Group II (semi-intensive system)	Autumn	Winter
Motility	120	40.17±0.69^B^	42.79±0.69^A^	39.70±0.67^B^	42.66±0.68^A^
Viability	120	48.95±0.65^b^	50.22±0.65^a^	48.52±0.66	49.60±0.65
Abnormality	120	13.39±0.32^a^	12.30±0.32^b^	13.73±0.32^a^	12.18±0.31^b^
HOS response	120	46.45±0.73	47.31±0.73	46.80±0.73	46.95±0.73
Acrosomal integrity	120	64.66±0.68^B^	69.30±0.68^A^	65.63±0.67^B^	68.34±0.68^A^

Means having different superscript in lower case letters (a, b) and upper case letters (A, B) in the row differ significantly at 5% (p<0.05) and 1% (p<0.01), respectively. SE=Standard error, HOS=Hypo-osmotic swelling

### Effect of season on semen freezability

The least square means of semen freezability parameters under different season of collection (autumn and winter) are presented in Table-2. The present study revealed that the effect of season significantly influences the ability of buck to withstand the freezing and thawing process. The spermatozoa from ejaculate collected during autumn were more cryosensitive than those collected during winter. The PTM and acrosomal integrity of spermatozoa were significantly (p<0.01) a higher in winter as compared to winter season. However, abnormalities in spermatozoa were significantly (p<0.05) higher in autumn season but percent viability and HOS response of spermatozoa did not differ significantly between the seasons. The effect of seasonal variation in post-freezing seminal characteristics could be attributed to change in melatonin and testosterone secretions [24]. The better freezabilty of boar semen collected during winter and spring than in summer and autumn in term of motility, viability, and abnormalities [25]. The present results were also in agreement with Tuli and Holtz [26] who reported semen freezing during winter have a higher proportion of motile spermatozoa in Boer buck. However, the present findings were contrary to those reported by D’alessandro and Martemucci [27] in ram and Wang *et al*. [28] in Sannen bucks. The interaction effect of the management system and season were found non-significant for all semen freezability parameters in this study.

## Conclusion

It is concluded that both management system and season affect freezability of buck semen. Semen collected from bucks reared under semi-intensive management system was better in terms of post-thaw semen quality than intensive system. Moreover, semen collected in winter (December-February) had higher semen freezability as compared to autumn season (September-November). Therefore, it could be suggested that semi-intensive management system and winter season are more suitable for cryopreservation of buck semen over intensive system and autumn season, respectively.

## Authors’ Contributions

BR has planned the study. NK conducted the experiment and recorded the data. NK performed semen evaluation with the help of SKJ and CG. SAB and SDK performed statistical analysis. NK, AK and SC drafted and revised the manuscript under the guidance of BR. All authors read and approved the final manuscript.
